# On the unraveling of ‘revitalization of local health traditions’ in India: an ethnographic inquiry

**DOI:** 10.1186/s12939-018-0890-1

**Published:** 2018-11-23

**Authors:** Arima Mishra, Devaki Nambiar

**Affiliations:** 1 School of Development, Azim Premji University, Bangalore, India; 2 Health Systems & Equity, George Institute for Global Health, New Delhi, India

**Keywords:** Local health traditions, Revitalization, Ethnography, India, Legitimacy, Inequities, Documentation

## Abstract

**Background:**

India has recently renewed emphasis on non-allopathic systems of medicine as a means to address the health needs of its populace. Earlier in 2002, its national health policy had sought to ‘revitalize’ community-based health knowledge and practices – jointly christened ‘local health traditions’. Yet policy texts remain silent on the actual means by which ‘revitalization of local health traditions’ should take place. Our research sought to understand the policy lessons *of* and *for* revitalization of local health traditions in the three Southern Indian states through an ethnographic inquiry in 2014–2016.

**Methods:**

Our inquiry included a narrative synthesis of policy texts tracing the history of governance processes and mechanisms pertaining to traditional medicine, including local health traditions, linking this to the activities of non-governmental organizations (NGOs) and networks involved in “revitalization”. Through in-depth interviews, observations and case studies, we sought to understand the life worlds of local health tradition practitioners and what revitalization meant to them. Our method revealed that beyond a purely academic inquiry, we needed an (inter)action that would give greater voice to these perspectives and views leading to hosting an interactive dialogue among practitioners, NGO representatives, academics, and government officials.

**Results:**

Our ethnographic inquiry unraveled the problematic of a litotic approach to local health traditions as those which are *non-* institutionalized, *non-*certified, *non*-documented; assuming the state to be the only source of power and legitimacy. Revitalization discussions were restricted (and often misled) by such an approach. Local health practitioners and others directed us to interesting possibilities of revitalization either through participatory modes of documentation of traditional health knowledge, strengthening existing collective forums for formal social recognition, and building pedagogical institutions that promote experiential learning.

**Conclusion:**

Were we not enabled by ethnography as a method that changes its shape apace with emerging findings, we would have not been able to comprehensively answer our questions. This is critical because not only was this already a marginalized area of inquiry, but with any other method we risked reinforcing inequities by imposing epistemological and other hierarchies on our participants– whom we would argue were partners - in arriving at our conclusions.

## Background

I believe that the strong calls we are hearing for a renewal of primary health care create an ideal opportunity to revisit the place of traditional medicine, to take a positive look at its many contributions to health care that is equitable, accessible, affordable, and people-centred (Director General, World Health Organisation [WHO] at the Congress on Traditional Medicine, 2008, Beijing, China) [1].

On the heels of this announcement by the former Director General of the WHO and subsequent Beijing declaration on traditional medicine [2], the 2009 World Health Assembly called for the upgrading of the WHO’s first traditional medicine strategy (2002–2005) to support member states in “harnessing the potential contribution of Traditional Medicine to health, wellness and people-centred health care; and promoting the safe and effective use of Traditional Medicine by regulating, researching and integrating Traditional Medicine products, practitioners and practice into health systems, where appropriate” [3]. This culminated in the launch of the WHO’s next Traditional Medicine Strategy (2014–2023) in the year 2013.

India’s policy prescriptions in this domain over the past two decades have followed this arc of global trends. In 2002, the first ever national policy on Indian Systems of Medicine and Homeopathy was passed. This policy candidly acknowledged the long neglect of state support for traditional systems of medicine including household and community based health knowledge and practices. Thus, along with the traditional systems of medicine like Ayurveda, Siddha, and Homeopathy, this policy, for the first time, recognized the contribution of folk medicine/community health knowledge and practices. The National Rural Health Mission (NRHM), the flagship program of the Government of India (later rechristened the National Health Mission (NHM)), offered a major boost to the spirit of strengthening traditional systems of medicine including folk medicine through concrete programmatic strategies starting in 2005. It proposed the twin strategy of *mainstreaming* traditional systems of medicine namely AYUSH (Ayurveda, Yoga & Naturopathy, Unani, Siddha, Sowa Rigpa, and Homeopathy) and *revitalizing* Local Health Traditions (LHT) as part of its overall mandate to strengthen the Indian public health system in rural areas. Policy discussions in India occurring around the time of the strategy’s revision, have called for, among other measures, documentation, validation and promotion of home and community based knowledge and practices including tribal medicine [4–7].

In 2014, the AYUSH division in the Ministry of Health and Family Welfare got its own ministry and the National AYUSH Mission was launched [8]. With the launch of International Yoga Day a year later, India signaled an emphasis on non-allopathic systems of medicine as a means to address the health needs of Indians as well as the global community. Along with several measures to strengthen traditional systems of medicine through research, training and practice, the recent 2017 National Health Policy additionally calls for developing mechanisms for certification of “prior knowledge of traditional community health care providers and engaging them in the conservation and generation of the raw materials required, as well as creating opportunities for enhancing their skills” [9]. (See Table 1 for a summary of the policy developments on traditional systems of medicine since 2002 onwards).

**Table 1:** Timeline of policy developments on traditional systems of medicine (since 2002)

Year	Policy developments	Key feature pertaining to traditional systems of medicine
2002	National Policy on Indian Systems of Medicine and Homeopathy	Acknowledged long neglect of traditional systems of medicine; revitalization of folk medicine mentioned for the first time
2005	National Rural Health Mission	Suggested mainstreaming of AYUSH and revitalizing local health traditions as part of strengthening primary health care
2014	Separate ministry of AYUSH formed Launch of National AYUSH Mission	To ensure optimal development and propagation of AYUSH systems of health care including LHT
2015	Launch of International Yoga Day	Promotion of yoga towards holistic health and wellbeing
2017	National Health Policy	Access to assured AYUSH services and support for documentation, validation and promotion of LHT

In our view, the significance of these policy developments is two-fold. First, the potential of AYUSH in achieving national health goals and thus its integration in the national health system, has received sharper attention. Second, ‘non-systems’ of medicine or community based health knowledge and practices, have found space in the state policy documents where they are acknowledged as having potential to contribute to strengthening primary health care. This recognition marks the coinage of the term ‘local health traditions’. Such traditions are defined as the *undocumented* knowledge (or folk health traditions) possessed by birth attendants (*dais*), bone setters, herbal healers, poison specialists etc. [10].

The recognition of local health traditions in policy documents is an important development in the history of health governance in India. In the organization of India’s health systems, traditions (such as folk medicine, indigenous healing) have had no clear legitimate place. Except for sporadic attempts at involving the providers of community based health knowledge and practice in community development programs through appropriate (re)training [11, 12], these traditions could not conform to centralized state governance instruments of professionalization, licensing, certification and standardization [13–15]. Yet, local health traditions have continued to be practiced among communities in different parts of India even at the margins of the state [13, 16–20]. A recent study showed that more than 80% of households in 14 out of 18 Indian states studied, reportedly utilized some form of local health tradition to treat episodes of minor illnesses (in the 3 months preceding the survey), in addition to its use in preventive and promotive health [21]. Select non-governmental and grassroots organizations (NGOs) and networks have contributed to nurturing such traditions with varying degrees of success [22–24].

The state’s recent turn towards such community based health traditions, at least in policy pronouncements, is connected to its plans for reorientation of health care delivery in meeting the national goal of universal access to health. The revitalization agenda in the NRHM was located within government’s overall promotion of comprehensive primary health care, that sought greater community ownership of health, in line with reviving the spirit of Alma Ata declaration. This fit with other community–based measures being implemented, like recruitment of a village level health activist, village level planning committees, community monitoring, and more [25, 26]. Revitalization of the NRHM, as part of a process to reach the goal of universal health care, also occurred within an Indian health context of persistent health inequity, growing double burden of diseases and high out of pocket expenditure [27, 28].

We find that the policy intent to revitalize local health traditions is critical and laudable, yet major policy texts do not elaborate on how these could be revitalized. In the twin mainstreaming-revitalization strategy of the NRHM as well as in subsequent AYUSH mission document, there are detailed guidelines on how AYUSH can be mainstreamed and strengthened. However, both these documents are silent on the actual means by which ‘revitalization of local health traditions’ is/ought to occur. An analysis of the NRHM’s strategies on the status of AYUSH and LHTs shows that while different states have innovated and translated the NRHM intent in mainstreaming AYUSH, very few states had much that is substantial regarding revitalization of LHTs for effective integration into the formal health systems delivery [29]. This first ever evaluation report of the NRHM twin strategy of mainstreaming-revitalization, noted that “Local Health Traditions, which have been ignored by most state plans, need to be incorporated within a conceptualization of the health care system so that they can be appropriately supported by state planning. They are autonomous forms of self-care and the initiation points of locally accessible primary health care that can be promoted through a few simple activities by the rural health service system” [29:08]. Research has further been undertaken to assess the effectiveness and/or map the processes of mainstreaming of AYUSH, elucidating appropriate policy recommendations for their strengthening but no study has unpacked the ‘what’ and ‘how’ of the revitalization of the local health traditions to inform policy development and implementation [30–32].

Our research was conceived as an opportunity to understand the (silences around) policy lessons *of* and *for* revitalization of local health traditions in the three Southern Indian states of Kerala, Karnataka, and Tamil Nadu in 2014–2016. This paper discusses the findings on such policy lessons and elucidates how an ethnographic inquiry enabled us to arrive at these findings.

## Methods

### Overview of the ethnographic research process

Our research relied on an ethnographic inquiry that broadly defines ethnography as “combining research design, field work and various methods of inquiry to produce historically, politically and personally situated accounts, descriptions, interpretations and representations of human lives” [33]. Following this definition and the work of other medical anthropologists, we wish to highlight three critical features of an ethnographic inquiry that we incorporated into our research process. First, it interrogates the framing of the ‘problem’ itself, situating it in the historical, political and societal contexts [34–37]. It is thus “attentive to the processes, structures and power relations that constitute the field in which a policy is both constructed and negotiated” [38]. Second, considering the complexity of social reality, ethnography commits itself to be responsive to multiple perspectives and their lived realities. The third underscores critical reflexivity as an integral component of ethnographic inquiry. It is hence necessarily mindful of self and others (researcher’s own positionality and that of the research participants), of interpretations of diverse (often contested) viewpoints, and research process that is ‘emergent, spontaneous and dialogical’ [34]. (See Table 3).

Our methodology – comprised of various methods (see Table 2) - was decidedly iterative and dynamic as distinguished from typical “one-time” qualitative research methods. Our research also drew a distinction, as indicated by Nichter, between “qualitative research” involving one time interviews, surveys, or focus groups, and ethnographic research that examines behavior and knowledge production as context sensitive and subject to contingencies and power relations as well as being performative and “multi-vocal” [39]. It embodied the latter, as Table 3 explains.

**Table 2:** Summary of methods

Method	Sample and field procedure
a) narrative synthesis of policies	22 policy documents at the national level as well as key international policies that were contemporaneous to or are reflected in the terms and concepts used in national policies.
b) stakeholder landscaping	Visits to and interactions with organisations and agencies in all three states, both referred to and indicated in publicly available policy documents on LHT, and as nominated by those interviewed
c) key informant interviews with NGO staff	Interviews carried out with 18 NGO representatives involved with the revitalisation agenda as indicated in policy documents or as referred by prior key informants
d) observations	Participant observations in meetings of 6 national, regional, and state-level healers associations and conclaves, as well as 5 scientific conferences, seminars and meetings
e) focus group discussions	3 discussions with convenience samples of healers at aforementioned conclaves to discuss what they do, why they attend these meetings and what they feel ought to be done to improve their situation
f) in depth interviews	Interviews carried out with 51 healers and 15 of their patients, 20 government representatives of AYUSH department as well as AYUSH research councils at the state level, 15 academicians/researchers involved with documentation efforts or broader research/writing/advocacy on LHT in the popular media or academic literature
g) interactive dialogue	1.5 day long interaction involving 36 of the aforementioned stakeholders in a direct conversation with each other on themes emanating from earlier fieldwork, i.e. a) documentation, b) linkages between LHT and AYUSH, c) recognition and legitimacy and d) ways forward for research, advocacy and policy.
h) case studies of healers	Repeated interviews carried out to develop case studies of 10 (6 menn and 4 women) healers to more deeply understand their experience in light of themes emerging from the dialogue. Care was taken to ensure diversity in gender, years of experience and representation of both those present and absent from dialogue

**Table 3:**
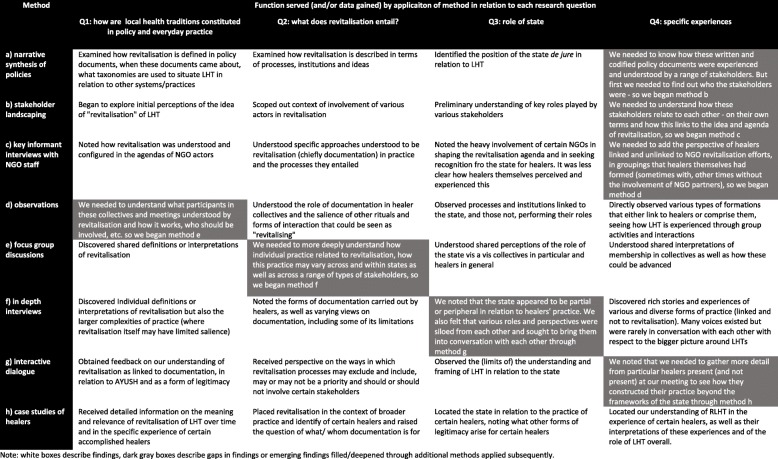
Summary of methods applied, findings generated and progression through methods.

Note: white boxes describe findings, dark gray boxes describe gaps in findings or emerging findings filled/deepened through additional methods applied subsequently

Source: Authors

In seeking to operationalize such an inquiry, our research began with interrogating the notions of ‘revitalization,’ and ‘local health traditions’ – seeking to understand the history and politics shaping both. We thus asked four questions:

1. ‘How are ‘local health traditions’ constituted in the policy frameworks and in their everyday practices?
2. What does revitalization entail?
3. What is the role of the state in relation to non-state actors including NGOs, community of healers in revitalization?
4. What are the specific experiences with revitalization of local health traditions including identifying opportunities and challenges across multiple perspectives – governmental agencies and departments, NGOs and the community of healers themselves (whose knowledge is sought to be revitalized)?

These questions required that our tools elicit perspectives and experiences of policy makers, officials in NGOs, government departments as well as the healers across multiple sites across the three states in southern India including Kerala, Karnataka and Tamil Nadu (see Table 2). We chose these states because of our prior knowledge of policies, institutions and practices related to local health traditions.

### Data collection process

We began with a narrative synthesis of national level health policy documents (health policies and reports of committees and task forces set up specifically on traditional medicine, *N* = 22)) seeking to understand the taxonomies and contexts of the rationale for revitalization starting with the first National Policy on Indian Systems of Medicine and Homoeopathy and the notion of revitalization of ‘local health traditions’ was made explicit.

Following from this policy analysis, we mapped NGOs and their networks working in the area of traditional medicine and local health traditions in the three states where our fieldwork was situated. We carried out multiple interviews with key informants in these organizations, observed their activities (documentation, relevant meetings) in the field sites and analyzed the organizational documents (eg: methodology for documentation of local health traditions, outcomes of documentation in the form of books, CDs, pamphlets, protocol for certification of healers, internal evaluation reports of organization’s efforts in revitalization) shared with us. Our interactions then extended to representatives of government departments and institutions specifically Department of Environment and Forests, AYUSH, government research councils on traditional medicine, and state Bio-diversity Boards to elicit their perspectives on opportunities and challenges in revitalization of local health traditions.

We then sought to understand the life worlds of *vaidyas* (what local health tradition practitioners are called), seeking to understand what revitalization meant to them in the context of their everyday practice. This happened over multiple interactions and observations of their practice including accompanying them to the forest, village health camps, preparation of the medicines in their homes and/or dispensaries. Our methodology allowed us to follow events, people and places – thus observing the Siddha Marma conference in Kanyakumari in Tamil Nadu with 300 healers to the Government and non-government organizations that led us to healers already working on documenting their knowledge. We conducted FGDs with healers in these collective forums.

Many months into fieldwork, our method revealed that beyond a purely academic inquiry, we needed an (inter)action that would bring these different perspectives together. This was important for three reasons. First, beyond just presenting different perspectives on revitalization of local health traditions, our research sought to provide a space for diverse perspectives to coalesce and dialogue with each other to see whether a shared understanding of revitalization would/could emerge. Second, we were mindful of the need to give adequate voices to the *vaidyas* – their worldviews, knowledge frames and their experiences in their own language- on whose behalf revitalization debates and discussions were being held everywhere. Third, we wanted to validate the direction of our enquiry. On 20-21January 2016, we hosted one and a half days interactive ‘dialogue’ among traditional healers/*vaidyas*, NGO representatives, academics, and government officials. The dialogue brought multiple perspectives, actors and experiences alive when key issues around recognition and legitimacy, documentation and future directions of local health traditions were debated and discussed. It also placed us as potential catalysts in a shared journey of advocating for meaningful revitalization. This was evident in a wrapping up session in this dialogue on ‘The Way forward’ where expectations and responsibilities on/for different actions on revitalization were spelt out for us.

This turning point in our research allowed both presentation and sharpening of our analysis. It gave direction to additional interviews and observations, specifically to explore the dimensions of gender and ethnicity (tribal/non-tribal healers). It also led us to follow up on documentation process by select healers and to understand further the scope of associations and networks in revitalization, as some of these were highlighted during the dialogue. We developed ten case studies of healers with different expertise, gender and nature of training by following each for a period of a week to ten days. This allowed a deeper understanding of what local health traditions constituted, modes of acquisition of knowledge, family repositories, interactions with patients, the evolving nature of the knowledge and practice, perceived challenges to the continuation of such practice, as well suggested possibilities of revitalization.

Data were collected over a period of eighteen months by the research team from January 2015 to June 2016. The two authors were the primary investigators. Three senior research associates, proficient in English and local languages in these three states (Kannada, Tamil and Malayalam) and familiar with this thematic domain were involved in data collection along with the two authors. We interviewed senior government officials, representatives of NGOs and academics. The research associates had prior training and experience in doing qualitative health systems research. They were supervised by the two authors, who are trained in anthropology and are experienced in ethnography methods. Apart from getting a weekly update on each of the field sites (shared with the authors by each of the research associates) there was a monthly skype meeting among the research team. This meeting discussed the progress, key insights and reflections on the processes of data collection and analysis, for all the three field sites during the entire data collection and analysis phase. Interview guides were prepared collaboratively by the research team following the narrative synthesis and the mapping of organizations exercise, which had helped us identifying key stakeholders and an overall thrust of their work. These guides evolved thematically and were modified, as we did the interviews, to speak to the different category of participants (government, NGO representatives, academics, healers),. Each interview was transcribed and translated into English soon after the data collection, and was discussed between the research associate and the authors. Key themes of each interview were noted on the transcript, for discussion during the monthly meeting.

### Data analysis

Data were analyzed concurrently, as they were collected through a process of open coding and thematic analysis. This concurrent analysis allowed, where necessary, for follow up interviews to better understand and situate the data. Specific attention was given to the process of local usage and context of the terms for local health traditions, legitimacy/recognition that repeatedly emerged as the themes of discussion. Transcripts of interviews with each category of respondents (healers, Government representatives, NGOs and their networks, academic/researchers) were arranged and analyzed separately for each state and then across states. This was followed by juxtaposition of these perspectives across categories weaving with the findings of the narratives synthesis, reports of observations of events, detailed proceedings of the interactive dialogue as well as secondary literature. Four analysis meetings, spanning two days each, were held. During these meetings, the entire research team discussed and finalized the findings of the study. Gleaning the experiences, interpretations, models and lessons of revitalization across states, led us to a more nuanced understanding of what revitalization means, what it entails, and what roles are played by various stakeholders in revitalization, definition, and practice of LHT.

## Results

This paper focuses on the shared findings across sites in relation to our research questions.

### LHT in national policy frameworks and in everyday practices

Originally drawn from the Greek word *litotes* means simple. It also means an understatement in which an affirmative is expressed as the negative of the contrary [40]. Our ethnographic inquiry unraveled an important and hidden reality of health care in India- revealing the problematic of a litotic approach to local health traditions as those which are *non-* institutionalized, *non-*certified, *non*-documented. Such a litotic reference emanates from the power of the state to define legitimacy of a system of medicine (and hence its inclusion in the national health systems) through standardized governance instruments of training, certification, registration and licensing. Our narrative synthesis showed how the organization of health services in post-independence context created a hierarchy of legitimacy with biomedicine at the top, followed by six Indian systems of medicine (later renamed as AYUSH in 2005), with ‘non-systems’ of medicine like local health traditions (earlier known as folk medicine/indigenous healing) as the ‘residua’ that fell outside the purview of the state. The recent coinage and turn to local health traditions (since 2002), continues to refer to these as undocumented, non-certified and non-institutionalized forms of knowledge and practice (as those that are not non-allopathy and non-AUSH). The Government as well as the non-government organizations, we spoke to, largely subscribed to such a view, manifest in their revitalization strategies.

*Vaidyas*/Healers, on the other hand, drew our attention to what local health traditions are, through the constitution of its knowledge base and practice. Local health traditions, as the healers explained, are learned and practiced through rigorous modes of knowledge acquisition and transmission. Our study focused largely on healers with specialized knowledge (*Vaidya* title is referred to healers with specialized knowledge only). Yet, the healers and key informants in the non-government organizations involved in revitalization, noted that certain local health knowledge was embedded within households and employed in everyday life in curative, preventive and promotive care. Specialized LHT knowledge, as explained to us by our participants, called for everyday practice of a different sort. For a specialized LHT healer, practice comprises everyday observations, doing and learning that includes knowledge about plants, their growth, modes of plucking and replenishment, preparation of medicines and their dispensation along with other lifestyle modifications. These experiential modes of learning necessarily involve extensive use of senses including smell, taste, touch of different plants and their therapeutic properties. Healers pointed towards the composite nature of such knowledge that goes beyond medicine or treatment, linking local ecology to nutrition and wellness in preventive and promotive health and even spiritual balance. The modes of learning and practicing entails specific qualities including sincerity, commitment, perseverance and passion for such knowledge and practice. Such qualities are highlighted as important constituents of local health traditions that have a service (*seva)* orientation distinguishing itself from a health care model that is driven by profit. Family lineage (*parampara*), as the site for a rigorous mode of learning and practicing, becomes an important marker of legitimacy for local health traditions.

Our study also revealed that LHT – far from being a unified category – as projected in the policy texts and implied in the revitalization strategies of the organizations we spoke to, represent a multiverse of healing experiences, expectations (from the state), modalities of revitalization (across gender, and types (tribal/non-tribal) of healers), modes of acquisition of knowledge (*paramparika* or traditional *vaidyas* and *non-paramparika vaidyas* including *nattu* or local *vaidyas*), and properties of healing expertise. Further, in different states, emphasis was placed on different dimensions of LHT in connection with practice. For example, in Kerala LHT practitioners were referred to as *paramparika nattu vaidyas* (traditional indigenous healers) emphasizing the lineage mode of transmission of knowledge and practice, while in Tamil Nadu, they are identified as *siddha vaidyas* (practitioner of siddha medicine) seeking to draw attention to the primordial nature of this practice to the institutionalized siddha medicine. In Karnataka, this meant *paramparika vaidyas* (traditional healers) or *gram vaidays* (village healers). These terms of reference are important as these draw attention to the legitimacy accruing from family lineage, local community distinguishing itself from quackery.

### Documentation as revitalisation of LHT

The predominant approach to revitalization whether sanctioned by the state or preferred by NGOs with funding from a variety of sources, was documentation [41]. The policy texts, we analyzed, cited various rationales for documentation including preservation due to threat of erosion of such knowledge (due to the apprenticeship mode of transmission and the perceived lack of interests among the younger generations towards such mode of learning), promotion of best practices among the community for preventive and promotive care through documentation and validation, potential for drug discovery as well as protection from probable commercial exploitation of such knowledge. Our study found that several documentation efforts were underway by AYUSH institutions, NGOs, university departments (botany, forestry, pharmacy among others) and research collectives. While mapping these documentation exercises, our research focused on interrogating the ‘who, what and how’ in documentation, to assess the viability of documentation as a mode of revitalization. Healers trained in family lineage, who were highly successful in their practice and had a large clientele, were not entirely convinced about the narrative of the threat of extinction of such knowledge and the urgent imperative to document. No one seriously contested the need to document local health knowledge. Yet, those healers involved in documentation shared concern about the lack of clarity of objectives of documentation; the ethnobotanical/ethno-medicinal nature of documentation assuming local health traditions to be about medicinal plants alone; as well as reducing knowledge holders/practitioners to mere informants in documentation surveys.

Healers participating in the dialogue and in our interviews ubiquitously felt that they were themselves were important – and neglected - stakeholders in the documentation:

Those who practice the knowledge need to be involved in documentation. How can someone who does not know the context and has never practiced the knowledge even understand what it is all about and document? *(*IDI_HEL_21_KA*).*

Documentation of local health knowledge cannot be everybody’s business. Someone who is sincere, dedicated and who has a respect and passion for such knowledge can and should document. It should not go into the hands of those with selfish motives (IDI_HEL_11_KE).

This concern is related to what is being documented which indeed delimits the scope of LHT in the form of registers of plants and remedies. Healers stated that LHT needed to be documented as community based health knowledge and not only by botanic properties or nosology of disease. There was an uneasy tension between LHT across these forms. On the one hand, documenting LHT was typically done in a format capturing the immediate and tangible forms of knowledge in terms of products and preparation drawing from methodologies employed by AYUSH-focused institutes and NGOs. Aspects that sometimes got excluded or ignored were workship and invocation of the God of forest (*Vana debta*) is as important as preparation and dispensation of herbs. In this view, the forest, as the source of healing properties (medicinal herbs) was sacred, as collection of plants was integral to conservation and replenishment in LHT practice. There were elaborate rules about modes, timing, techniques of collection of herbs and their preparation, which the healers shared with us in general terms that they felt were integral to understanding, acquiring and recording knowledge. The rules pertain to detailed dietary and other lifestyle prescriptions that they perceived as critical to the process of healing. The healers therefore contested the narrow and confining vision of documentation. Documentation with a focus on medicinal plants (in terms of botanical names, medical usage and application to ailments) was but a first step that should not be exclusive of the social context of the use of LHT knowledge.

Participants felt that documentation must be linked to practice; without which it may lead to mere museumization of knowledge (stored in libraries and filing cabinets) –having the opposite effect of “revitalization.” Officials spearheading such documentation effort conveyed to us somewhat wistfully:

We have collected local knowledge on health and medicine but we do not know what to do with this. Several files of such knowledge are stored in the cabinet quite safely. Perhaps now they need to be validated? (IDI_GOV_03_KA).

Others including NGO representatives took things a step further with a view shared by all the healers we spoke to as well:

Documentation has real meaning when this [knowledge contained in the documentation] is actively promoted in the community. Thus, without the practice of such knowledge, mere documentation will have limited purpose (IDI_NGO_07_KE).

Most documentation efforts lacked a larger strategy or intent on what the process would lead to. Further, since the myriad of documentation efforts had multiple objectives, it would be difficult to have them cohere under such a broader strategy or intent. While some had the clear intent of discovering new drug formulation, for others, documentation aimed at active promotion of such knowledge for strengthening primary health care, yet some others found the documentation exercise itself to be a process of social legitimation of such knowledge. Each of these objectives demands different methodological process, involvement of actors and outputs. Healers we spoke to specifically raised concern about the lack of clarity of objectives and outcomes. The objective of drug discovery for potential commercial purpose, and promotion of primary health care through community ownership of health, are contrasting objectives. We came across three specific models of documentation that hold promise for a more inclusive process, in the spirit of NRHM. These were explained to us by key informants from the organizations who developed these models. According to them, these models relied on a bottom up approach with the local community and healers playing an important role in establishing the first tier of legitimacy of knowledge, healer and practice based on experiences of the community. The models involve documentation through the healers, in conversations with practitioners of institutionalized medicine (eg: Ayurveda) [42, 43]. Additionally, the dialogue drew attention to other documentation efforts that adopted the methodological approaches of active listening and cooperative inquiry. Such attempts sought to enter into the world views of traditional midwives or barefoot gynecologists, while recording their knowledge and elements of practice [22, 23]. The latter two models did not seek out to ‘document’ as such, instead they were more in the spirit of reviving and strengthening community based health knowledge and practice, in order to promote community ownership of health. The emphasis in these models of documentation is on the methodological process which relied on a dialogical approach. These documentation efforts while capturing the strengths of such traditions also pointed out areas where each tradition would need to be improved or strengthened. As the discussion in the dialogue made it apparent, a key challenge in documentation of LHT has been to capture the strengths of such knowledge in a language that is legible to more mainstream, systems-based medical knowledge and practice.

### Roles and possibilities for revitalization of LHT

While the state officials in our study were a bit cautious in terms of the specific role of the state in revitalization of the local health traditions, NGOs representatives preferred that the state act as a facilitator of ongoing efforts rather than attempting to mold LHT in the line of bio-medicine or institutional systems of traditional medicine. Healers and representatives of select grassroot organizations pointed out the limitation of documentation as the only or main model of revitalization as well as the danger of a state-led mode of regulating providers. They pointed towards three possibilities of community based revitalization, which envisioned a central role for the state, not as merely regulator but also as a facilitator.

The first possibility presented by community based revitalization was the strengthening of the already existing healers’ collectives and associations which bring together healers through conclaves and meetings providing a platform for exchange of knowledge, identify potential for further research as well as acting as a self-regulating body. In Tamil Nadu, such collectives have played an important role in continuing education and training of younger generation of healers, introducing a community health curriculum in formal institutions and building repositories of family based knowledge. In the state of Kerala, such collectives have sought to garner legitimacy through commendations for senior healers with long standing service to the community as well as evolving collaborative research with medical institutions. In Karnataka, healers’ associations have organized health camps, and experimented with introducing certificate courses through experiential mode of learning.

The second possibility, strongly indicated by healers, is that pedagogical institutions could promote experience based learning through close mentoring for such knowledge to be passed on to the next generations. For them, such mode of transmission of knowledge and practice is much more sustainable than documentation. Members of the Healers’ Association actively deliberated on this possibility in their meetings and conclaves that we attended. Models of *gurukula* based education do exist and in their view, offered promise.

The third possibility was documentation where healers were partners in the production and use of knowledge. Contrary to the commonly held notion that local health traditions are undocumented, several family based generational healers showed us the documentation of their knowledge and practice in regional languages and dialects that are used for their everyday practice. They also shared that these documents evolve with new knowledge, emerging health ailments and practice and hence are not static or closed. They raised the issue that documentation needs to speak to different kinds of audiences, including the community, the healers, and the institutionalized systems of medicine.

## Discussion

Ethnography, is increasingly being seen as an important methodological lens in health policy and systems research [38, 44–47]. Our study showed why a critical ethnographic stance was important to be able to ask a set of deeper questions about the policy on local health traditions and their revitalization, that focus more squarely on the lived experience of policy and on its operationalisation. Ethnographic inquiry is well positioned to allow asking of awkward questions and to approach core policy concepts with some criticality [34–37]. We sought to unpack the meanings, contexts and interpretations of ‘local health traditions’ and their ‘revitalization’, both of which find mention only in very recent policy texts. We found in our interviews, that neither LHT nor revitalization are self-evident categories. Understanding the time, context and processes that have brought about an otherwise marginal body of knowledge to the forefront through the coinage of local health traditions was important as no research on local health traditions can afford to be oblivious to the power asymmetry in which such traditions are nested.

The term of reference for LHT in state policy documents in litote as non-documented, non-system etc. signified a kind of power asymmetry. It also assumed a role for revitalization through particular and preset forms of documentation, certification and institutionalization. Our fieldwork revealed that the deployment of litotes in identifying local health traditions in fact simplifies a complex plurality of practices and persons. Revitalization discussions were restricted (and often misled) by such a documentation-centric approach, which did not yield an understanding of what the strengths (and limitations) of these traditions are. We were able to explore concrete possibilities (as well as specific challenges) of revitalization, by approaching LHT to understand what these traditions stand for, what the modes of knowledge acquisition and transfer were, how these are practiced, and what the sources of legitimacy are. Using this approach we privileged the perspectives of its practitioners.

Prior research has found that language and categories in international development, for example, traditional medicine, traditional birth attendants, as is discussed in the context of Nepal, efface local understanding and contextual translations of such categories [14]. In this context, the language of “traditional medicine”, and “traditional birth attendant” were cast as retrogressive in international development discourse serving on the one hand to make development institutions the locus of authoritative knowledge and on the other, completely devaluing local forms of knowledge [14]. In such a context, a development program that is meant to empower local communities only reinforces power asymmetry through the rejection of certain words and taxonomies. Local health traditions as a unifying category that brought together diverse set of practices though may have a significance in its visibility vis a vis the allopathic systems of medicine and AYUSH in the policy frameworks, had no meaning on the ground, beyond the policy texts. LHT existed in these three states as plural categories with varying internal logics and relationships based on local frames of reference as well as symbolic, political and pragmatic connotations. It is therefore somewhat arbitrary to assume a single policy treatment or frame for these traditions. Programs of revitalization will likely only succeed if they are designed in cognizance of such specificities.

One of the biggest tensions expressed in our data, emerged from the fact that votaries and practitioners of LHTs were concerned with practice whereas the state logic has been to categorize by system, resulting in the grouping of diverse LHTs into the single category of non-system. This results in a single, homogenous category being created that may not only be inapplicable for the diversity of practices represented, but also poses a larger threat to the very agenda of revitalization. Our research findings reinforced how critical it is to understand local interpretations and contextual usages of terms and categories and to be sensitive to the threats posed by imposing and homogenizing categories [14, 34, 48].

Considering the complexity of policy processes, capturing multiple perspectives and experiences is central to the field of HPSR. An ethnographic inquiry allows one to draw out these different perspectives across different sites and locales of power. It also allows for the creation of a dialogue or confrontation between these perspectives and spaces through which a ‘deeper understanding of the larger picture becomes possible’ [34, 37]. Our study provided an interactive dialogue space, where these perspectives and experiences on revitalization of local health traditions debated, clarified and coalesced, drawing and redrawing power hierarchy among the stakeholders. This dialogue began to question the linearity and certainty of the policy prescription on revitalization of local health traditions (through documentation, validation and promotion and/or certification of healers) and brought to the fore tensions and challenges in going about revitalization and identifying the role of different stakeholders. In the acknowledgment of and confrontation with these tensions, deeper conversations arose about who, what and how, of effective and meaningful modes of documentation. Future conversations need to consider different kinds of documentation - family repositories of healers as well as those that systematically record such knowledge and practice through participatory methodologies. This is an area that calls for more collaborative and trans-disciplinary thinking, rather than the siloes or vertical approaches in use thus far [23, 49].

Our critical ethnographic inquiry was sensitive to the hidden, invisible spaces as well as the visible. It uncovered the need for reorienting the lens to approach local health traditions (seeing from the perspectives of the practitioners of such traditions) to understand its strengths, scope and challenges in revitalization. It also uncovered that documentation as a mode of revitalization can be made more meaningful through a dialogical process. Our inquiry further led to identifying the less obvious possibilities of revitalization (beyond documentation). These included strengthening healers’ associations and collectives and reviving pedagogical institutions (in the style of the older *Gurukul* system) to nurture experience based learning. Evidence in others contexts demonstrates the potential of healers’ associations to not merely accrue social recognition to healers but also to contribute to strengthening the practice of such traditions in the provision of primary health care [50]. If the revitalization mandate seeks to strengthen community ownership of health (as enunciated in the NRHM policy), it is important to carefully nurture community based institutions like the healers’ collectives.

## Conclusion

Recent policy developments promoting the role of non-allopathic systems of medicine, specifically local health traditions, in strengthening primary health care have been significant. This is critical considering India’s current race towards achieving health for all. Our policy analysis began to reveal which policy measures could achieve such objectives, but not how. Our ethnographic inquiry revealed that far from a linear translation of policy measure, a critical, reflexive methodological engagement could unpack the meaning, contexts and interpretations of revitalization of local health traditions to break the silence (in policy documents) on the specific ways to operationalize the policy intent. Were we not enabled by ethnography as a method that changes its shape apace with emerging findings, we would have not been able to as comprehensively answer our questions about the policy lessons for revitalization of local health traditions. This is critical because not only was this already a marginalized area of inquiry in health research, but with any other method we risked reinforcing inequities by imposing epistemological and other hierarchies on our participants– whom we would argue were partners - in arriving at our conclusions.

## Abbreviations

AYUSH: Ayurveda Yoga Unani Siddha Homeopathy
FGD: Focus Group Discussion
LHT: Local health traditions
NGO: Non-Governmental Organization
NRHM: National Rural Health Mission
WHO: World Health Organization
